# Survivin Is a Central Mediator of Cell Proliferation in HPV-Negative Head and Neck Squamous Cell Carcinoma

**DOI:** 10.3390/cancers17172864

**Published:** 2025-08-31

**Authors:** Jing Zhu, Jianhong An, Erqiang Hu, Gregory Rosenblatt, Gabriela Berner, Aadita Roy, Nicole Kawachi, Nitisha Shrivastava, Vikas Mehta, Jeffrey E. Segall, Michael B. Prystowsky, Thomas J. Ow

**Affiliations:** 1 Department of Pathology, Albert Einstein College of Medicine, Bronx, NY 10461, USA; jing.zhu@einsteinmed.edu (J.Z.); jianhong.an@einsteinmed.edu (J.A.); erqiang.hu@einsteinmed.edu (E.H.); gregory.rosenblatt@einsteinmed.edu (G.R.); gabriela.berner@einsteinmed.edu (G.B.); nicole.kawachi@einsteinmed.edu (N.K.); jollynitisha@yahoo.com (N.S.); vikameht@montefiore.org (V.M.); jeffrey.segall@einsteinmed.edu (J.E.S.); michael.prystowsky@einsteinmed.edu (M.B.P.); 2 Department of Otolaryngology-Head and Neck Surgery, Montefiore Medical Center, Bronx, NY 10461, USA; 3 School of Engineering and Applied Science, University of Pennsylvania, Philadelphia, PA 19104, USA; aadita@seas.upenn.edu; 4 Memorial Sloan Kettering Cancer Center, 1275 York Avenue, New York, NY 10065, USA

**Keywords:** head and neck squamous cell carcinoma (HNSCC), CDK4, CDK6, palbociclib, cell proliferation, survivin, LQZ-7i, ubiquitin specific peptidase (USP1)

## Abstract

Background/Objectives: HNSCC is a highly aggressive malignancy marked by the dysregulation of the cell cycle. In HPV^−^ HNSCC, mutations in the CDKN2A gene frequently result in the loss of the p16 protein, a key inhibitor of the cyclin D1/CDK4/6 complex. This loss results in unchecked G1/S phase progression. The CDK4/6 inhibitor palbociclib has shown therapeutic potential in HPV^−^ HNSCC by inducing G1 phase arrest and reducing cell viability. In this study, we investigated the molecular mechanisms by which palbociclib affects cell viability in HPV^−^ HNSCC. Methods: Four HPV^−^ HNSCC cell lines were treated with palbociclib, and RNA sequencing was performed to assess changes in gene expression. Cell viability was measured using the MTT assay. To further investigate protein localization, interactions, and function, we used immunofluorescence staining, co-immunoprecipitation, small molecule inhibitors, and siRNA-mediated knockdown. Results: We demonstrate that palbociclib downregulates survivin, a protein that plays dual roles in mitosis and apoptosis, thereby inhibiting cell proliferation. We also found that survivin is overexpressed in HPV^−^ HNSCC. Inhibiting survivin dimerization using the compound LQZ-7i significantly reduces cell viability and promotes its export from the nucleus to the cytoplasm. Additionally, we identified USP1, a deubiquitinase, as both a downstream target of CDK4/6 and a key regulator of survivin stability. Inhibiting USP1 activity or silencing its expression significantly reduces survivin levels. Conclusions: Our findings highlight survivin as a critical mediator of cell proliferation in HPV^−^ HNSCC and suggest that targeting the CDK4/6-USP1-survivin axis may offer a promising therapeutic strategy.

## Simple Summary

Human papillomavirus-negative (HPV^−^) head and neck squamous cell carcinoma (HNSCC) develops through multiple mutations in key tumor suppressor genes, most notably CDKN2A. These mutations lead to the loss of the p16 protein, an important inhibitor of CDK4/6. Palbociclib, a selective CDK4/6 inhibitor, has shown potential in treating HPV^−^ HNSCC by inducing G1 phase cell cycle arrest and reducing cancer cell viability. This study investigates the molecular mechanisms underlying palbociclib’s effect on cell viability. We demonstrate that palbociclib decreases cell viability primarily by inhibiting cell proliferation through the downregulation of survivin, a protein that plays dual roles in mitosis and apoptosis. Furthermore, we identify USP1, a deubiquitinase, as a molecular target of CDK4/6 and a critical regulator of survivin stability. These findings suggest that targeting survivin or its upstream regulators could enhance the therapeutic efficacy of CDK4/6 inhibition in HPV^−^ HNSCC.

## 1. Introduction

Cell cycle dysregulation is a key feature of head and neck squamous cell carcinoma (HNSCC). HNSCC can be associated with human papillomavirus (HPV) infection, with HPV-positive (HPV+) cancers exhibiting distinct characteristics and outcomes compared to HPV-negative (HPV^−^) cancers [1]. In HPV+ cancers, the viral E6 protein binds to and targets p53 for degradation, leading to the loss of G2/M checkpoint control. Additionally, the viral oncoprotein E7 binds to and targets Rb for degradation, causing the nuclear translocation of E2F and promoting the transition into the S-phase [2]. In HPV^−^ cancers, mutations in the CDKN2A gene frequently lead to the absence or dysfunction of the p16 protein, which disinhibits the cyclin D1/CDK4/6 complex. This results in the phosphorylation of Rb, releasing E2F and driving uncontrolled G1/S progression and excessive cell proliferation [3]. HPV^−^ HNSCC is generally more aggressive, associated with poorer prognosis, lower treatment sensitivity, and reduced overall survival rates compared to HPV+ HNSCC. As a result, there is a critical need for improved therapeutic strategies for patients with HPV^−^ HNSCC.

Palbociclib, a selective CDK4/6 inhibitor, is used for treating estrogen receptor-positive and HER2-negative advanced or metastatic breast cancer [4]. Previous research has shown that palbociclib treatment can induce G1 phase arrest and reduce cell viability in HPV^−^ HNSCC cells [5]. Combining palbociclib with radiation therapy could offer a novel treatment approach for HPV^−^ HNSCC [6]. However, the molecular mechanisms underlying palbociclib’s effect on cell viability remain unclear. In this study, we employed RNA sequencing to assess changes in gene expression following palbociclib treatment in HPV^−^ HNSCC cells and identified survivin, the smallest member of the inhibitor of apoptosis (IAP) family, as the key regulator in controlling cell proliferation.

Cell division and apoptosis are essential processes for development and tissue homeostasis, and any disruption in these processes can lead to uncontrolled cell growth, a hallmark of cancer and other diseases [7]. Survivin, also known as BIRC5, appears to play a role in both processes, acting as an apoptosis inhibitor and a mitotic effector [8,9]. This dual role has made survivin a prominent target in cancer research. The IAP family includes eight members: BIRC1/NAIP, BIRC2/cIAP1, BIRC3/cIAP2, BIRC4/XIAP, BIRC5/survivin, BIRC6/Apollon, BIRC7/ML-IAP, and BIRC8/ILP2 [10,11]. Survivin is unique in that it is largely undetectable in normal adult tissue under non-stress conditions but is highly expressed in transformed cells and various human cancers [12,13]. Its expression has been linked to chemotherapy resistance and tumor aggressiveness [14,15].

Survivin consists of 142 amino acids and contains two distinct domains: an N-terminal Zn^2+^-binding BIR domain and a 65 Å amphipathic C-terminal α-helix. The crystal structure of human survivin reveals a bow-tie-shaped dimer. In addition to its BIR domains, the dimer features two unusual C-terminal α-helical extensions, which may facilitate protein–protein interactions [16,17]. During mitosis, survivin localizes to the mitotic spindle, interacting with microtubules [18]. It associates with Aurora B kinase and the inner centromere protein in the chromosome passenger complex, which is crucial for proper chromosome segregation and cytokinesis [19]. To inhibit apoptosis, survivin interacts with various factors to prevent cell death [20]. With the help of cofactor HBXIP, survivin binds to and inhibits pro-caspase 9 [21], and in complex with XIAP, it suppresses caspase 9 [22]. Survivin can also influence caspase-independent apoptosis by inhibiting AIF [23]. The knockout of survivin using CRISPR/Cas9 in leukemic cells induces apoptosis and inhibits cell proliferation [24]. RNAi-mediated knockdown of survivin triggers apoptosis in neuroblastoma via mitotic catastrophe and leads to significant G2/M cell cycle arrest and impaired chemotaxis in vascular smooth muscle cells without causing cytotoxicity [25,26].

The CDK4/6-Rb pathway drives cell cycle progression, and the ubiquitin-proteasome system (UPS) regulates protein degradation and cell cycle control [27,28]. Survivin expression peaks during the G2/M phase of the cell cycle and rapidly declines in the G1 phase [18]. This temporal pattern suggests that both the CDK4/6-Rb pathway and the UPS might regulate survivin expression. However, the detailed mechanisms governing survivin regulation remain poorly understood.

In this study, we demonstrate that CDK4/6 inhibition with palbociclib in HPV^−^ HNSCC cells reduces cell viability by downregulating the expression of survivin. Moreover, we show that the subcellular localization of survivin appears to be linked to its function, and its upregulation in HPV^−^ HNSCC cells can be modulated through the activation of CDK4/6 and USP1, a deubiquitinase. Our findings suggest that targeting the CDK4/6-USP1-survivin axis may offer a promising therapeutic strategy.

## 2. Materials and Methods

### 2.1. Antibodies

Rabbit polyclonal antibody against USP1 (no. 14346-1-AP) was purchased from Proteintech Group (Rosemont, IL, USA). Mouse mAb against β-actin (no. 3700), rabbit mAb against Bax (no. 2772), Bcl-xL (no. 2764), Bim (no. 2819), Mcl-1 (no. 5453), and survivin (no. 2808) were from Cell Signaling Technology (Danvers, MA, USA). Rabbit polyclonal antibody against c-IAP1 (no. sc-7943) was from Santa Cruz Biotechnology (Dallas, TX, USA). Rabbit polyclonal antibody against Bak (no. 06-536) was from Millipore Sigma (Burlington, MA, USA). Alexa Fluor 568 donkey anti-mouse IgG (H + L) (no. A10037), Alexa Fluor 488 donkey anti-rabbit IgG (H + L) (no. A21206), Goat anti-rabbit IgG (H + L) cross-adsorbed secondary antibody Dylight 800 (no. SA5-10036), and Goat anti-mouse IgG (H + L) cross-adsorbed secondary antibody Dylight 680 (no. 35519) were from Thermo Fisher Scientific Technologies (Waltham, MA, USA).

### 2.2. Cell Culture

CAL27 and HN5 cell lines were obtained from the University of Texas MD Anderson Cancer Center Head and Neck Cell Line Repository (Houston, TX, USA), with appropriate material transfer agreements and confirmed and validated with STR genotyping. Both lines have been previously reported as HPV-negative [29]. Growth medium included high-glucose DMEM (Fisher Scientific, Waltham, MA, USA, no. 11965-092) supplemented with 10% fetal bovine serum (Life Technologies, Carlsbad, CA, USA, no. A5669402), 0.1 mM nonessential amino acids (Cytiva, Marlborough, MA, USA, no. SH30238.01), 1 mM sodium pyruvate (Gibco, Miami, FL, USA, no. 11360070), and 100 U/mL penicillin/streptomycin (Corning, Tewksbury, MA, USA, no. 30-002-CI). All cells were cultured within a humidified 5% CO_2_ at 37 °C.

CR15 and CR18 are HPV^−^ HNSCC cultures derived from patients and established at our institution using conditional reprogramming (CR) techniques [30,31]. The HPV-negative status of CR15 and CR18 were confirmed by RT-PCR targeting HPV16 E6 and E7 mRNA transcripts. CR cells were co-cultured with irradiated (30 Gy) 3T3 fibroblast feeder cells in F-medium at 37 °C with 5% CO_2_. The F-medium consisted of 75% complete DMEM and 25% Ham’s F-12 nutrient mix (Gibco, Miami, FL, USA, no. 11765–054), supplemented with the following: 250 ng/mL amphotericin B (Fisher Scientific, Waltham, MA, USA, no. BP264550), 0.125 ng/mL epidermal growth factor (Life Technologies, Carlsbad, CA, USA, no. PHG0313), 10 μg/mL gentamicin (Gibco, Miami, FL, USA, no. 15710–064), 10 μmol/L ROCK inhibitor Y-27632 (Enzo, Farmingdale, NY, USA, no. 270–333M025), and 0.1 nmol/L cholera toxin (no. C-8052), 25 ng/mL hydrocortisone (no. H-0888), and 5 μg/mL insulin (no. I-5500) from Sigma-Aldrich (St. Louis, MO, USA).

### 2.3. Drug Treatment

Cells were treated with drugs at indicated concentrations for 24 h or 72 h. Control cells were treated with an equivalent amount of vehicle (dimethyl sulfoxide or H_2_O). The following drugs were used: ML323 (no. S7529), LQZ-7i (no. E0108), and palbociclib (PD-0332991) (no. S1116) from Selleck Chemical (Houston, TX, USA).

### 2.4. MTT Cell Viability Assay

Cells were plated at 4000 cells per well in 96-well plates and exposed to different concentrations of LQZ-7i or ML323 for 72 h. Medium was removed and the MTT reagent (Sigma-Aldrich, St. Louis, MO, USA, no. 475989), 3-(4,5-dimethylthiazol-2-yl)-2, 5-diphenyltetrazolium bromide, was added to cells at a final concentration of 1 mg/mL. Plates were then incubated at 37 °C for 2 h in the dark. The supernatant was removed from the wells, and formazan crystals were dissolved in dimethyl sulfoxide. The Benchmark Plus microplate reader (Bio-Rad, Hercules, CA, USA) was used to measure optical density of the resulting solution at 570 nm.

### 2.5. Hemocytometer Cell Counting

CAL27 and HN5 cells were seeded at 1.2 × 10^5^ cells per well and 0.8 × 10^5^ per well, respectively, in 6-well plates and exposed to 7.5 µM LQZ-7i for 72 h. The untreated control cells were exposed to an equivalent amount of dimethyl sulfoxide (<0.1%). A cell suspension was collected, mixed with trypan blue (Gibco, Miami, FL, USA, no. 15250061), and then counted using the hemocytometer.

### 2.6. Western Blotting and Co-Immunoprecipitation

Cells were washed with ice-cold phosphate-buffered saline (PBS) (Corning, Tewksbury, MA, USA, no. 21-031-CV) and lysed in radioimmunoprecipitation assay (RIPA) buffer containing Pierce protease and phosphatase inhibitors (no. A32959) (Thermo Fisher, Waltham, MA, USA) for 30 min on ice. The homogenates were centrifuged at 14,000× *g* at 4 °C for 40 min to remove insoluble material. Protein concentration was estimated with RC DC protein assay kit (no. 5000121) (Bio-Rad Laboratories, Hercules, CA, USA). Cell lysates were mixed with 5 × Laemmli sample buffer containing 2-mercaptoethanol (Bio-Rad, Hercules, CA, USA, no. 1610710) and incubated at 95 °C for 5 min. The proteins (–30 μg per lane) were separated by 10% SDS-PAGE, followed by their transfer from the gel to a poly (vinylidene difluoride) (PVDF) membrane. The PVDF membrane was blocked with a 5% solution of nonfat milk and incubated at 4 °C overnight with primary antibodies (1:1000). Following a washing step to remove unbound primary antibody, we used goat anti-rabbit IgG (H + L) cross-adsorbed secondary antibody Dylight 800 and goat anti-mouse IgG (H + L) cross-adsorbed secondary antibody Dylight 680, both diluted 1:5000, and incubated them for one hour at room temperature. The LI-COR Odyssey Fc imaging system was used for detection, and LI-COR’s Image Studio Lite software (version 5.5) was used for analysis. Immunoprecipitations were performed using cell lysates, with the addition of the indicated primary antibody (anti-survivin at 1:250). After overnight rotation at 4 °C, the immunocomplexes were precipitated adding protein A (Thermo Fisher, Waltham, MA, USA, no. 17528001) agarose conjugates for an additional 3 h at 4 °C, then washed 3 times in HNTG buffer (prepared by our laboratory) and resuspended in 2 × Laemmli sample buffer (Bio-Rad, Hercules, CA, USA, no. 1610747). Samples were analyzed by Western blotting.

### 2.7. Immunofluorescence Staining

Cells cultured on glass coverslips were fixed using a 3% paraformaldehyde (Fisher Scientific, Waltham, MA, USA, no. J61899.AK) solution and permeabilized/blocked in PBS containing 2% donkey serum (Sigma Aldrich, St. Louis, MO, USA, no. D9663), 1% bo vine serum albumin (Fisher Scientific, Waltham, MA, USA, no.BP-9706-100), and 0.1% Triton X-100 (Fisher Scientific, Waltham, MA, USA, no. BP151-100) for one hour at room temperature. Cells were then incubated with primary antibodies (1:250) overnight at 4 °C. For visualization, cells were stained with Alexa Fluor 488 donkey anti-rabbit IgG (H + L) and Alexa Fluor 568 donkey anti-mouse IgG (H + L) fluorescent secondary antibodies, both diluted at 1:250, for one hour at room temperature in the dark. The nuclei were labeled with DAPI (4′,6-diamidino-2-phenylindole) (Sigma Aldrich, St. Louis, MO, USA, no. DUO82040). Images were captured under a Zeiss Axiovert 5 fluorescent microscope (Pleasanton, CA, USA).

### 2.8. Immunohistochemistry

The HNSCC tissue samples from 12 patients were obtained from Montefiore Medical Center with informed consent (New York, NY, USA). This study was approved by the Institutional Ethics Committee and Institutional Review Board of Montefiore Cancer Center (Reference Committee Number 2018-8778). HPV status was assessed using p16 immunohistochemistry staining. Formalin-fixed and paraffin-embedded specimens were cut into thin sections (4 µM) and attached to glass slides. The sections were deparaffinized in xylene (Sigma-Aldrich, St. Louis, MO, USA, no. 534056) and rehydrated through a graded ethanol series, followed by antigen retrieval with Tris-EDTA buffer (pH 9.0) (Novus Biologicals, Toronto, ON, Canada, no. NB900-62085) at 100 °C for 20 min. Endogenous peroxidase activity was inhibited using the peroxidase suppressor (Thermo Scientific, Waltham, MA, USA, no. 35000). Following the incubation with universal blocking buffer (Thermo Scientific, Waltham, MA, USA, no. 1859332), the slides were then incubated overnight at 4 °C with primary antibodies against survivin at a dilution of 1:250. Secondary antibodies used were anti-rabbit HRP-conjugated antibodies (Invitrogen, Carlsbad, CA, USA, no. 32460). Reactive products were visualized with 3,3′-diaminobenzidene (DAB) (BD Biosciences, Franklin Lakes, New Jersey, USA, no. 550880) as the chromogen. The slides were counterstained with hematoxylin (Sigma-Aldrich, St. Louis, MO, USA, no. H9627), dehydrated through a graded series of ethanol solutions, and cleared with xylene. Images were captured under a Zeiss Axiovert 5 fluorescent microscope (Pleasanton, CA, USA) at 400× magnification.

### 2.9. RNA Extraction and Next-Generation Sequencing

Cells were seeded in 60 mm dishes and treated with 1 µM Palbociclib for 24 h. Total RNA was extracted from four different cell lines using the RNeasy Kit (Qiagen, Hilden, North Rhine-Westphalia, Germany), with two independent replicates prepared per cell line. RNA libraries were generated simultaneously for all samples using the NEBNext^®^ Single Cell/Low Input RNA Library Prep Kit for Illumina (New England BioLabs, Ipswich, MA, USA).

Sequencing was performed on the Illumina NovaSeq X Plus system by Novagen Biolabs at the Albert Einstein College of Medicine. Reads were aligned to the Homo sapiens reference genome (hg38) using STAR (v2.4.0.1; RRID:SCR_004463). Gene expression levels were quantified using raw count values, and differential gene expression analysis was conducted using DESeq2 (v1.40.2; RRID:SCR_000154). Pathway analysis was performed using the Molecular Signatures Database (MSigDB) (https://www.gsea-msigdb.org › gsea › msigdb (accessed on 27 August 2025)).

### 2.10. Data Collection and Analysis

RNA microarray data for survivin/BIRC5 (ENSG00000089685.13) in lip/oral cavity HNSCC were retrieved from the existing data repository in the Department of Pathology at the Albert Einstein College of Medicine (AECOM), from gene expression microarray studies of HNSCC tumors as previously reported [32,33]. There were 44 pairs of primary tumor and solid normal tissue samples. (Note: “solid tissue normal” are samples taken from normal mucosa tissue tissue near the tumor site). For statistical analysis, we employed the stats library to perform Wilcoxon Rank Sum and Signed Rank Tests. For survival analysis using Kaplan–Meier curves, we utilized the survival and survminer libraries.

## 3. Results

### 3.1. The CDK4/6 Inhibitor Palbociclib Suppresses Survivin Gene Expression

**Figure 1:**
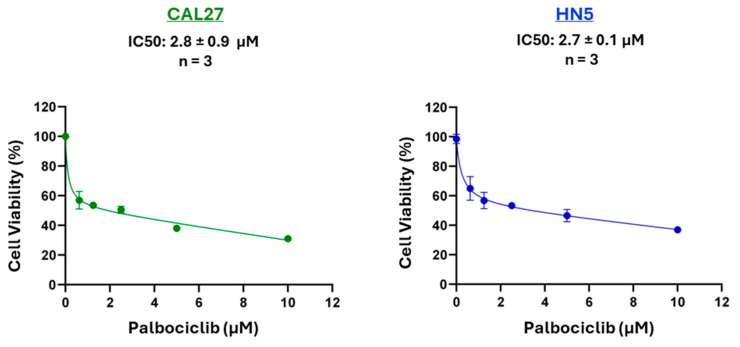
Effect of palbociclib on cell viability. CAL27 and HN5 cells were seeded in 96-well plates and treated with palbociclib at specified concentrations for 72 h. An MTT assay was performed, and representative graphs are shown. IC50 values represent the means ± SEM from three independent experiments conducted in triplicate.

Palbociclib is a selective inhibitor of the cyclin-dependent kinases CDK4 and CDK6. Previous studies have demonstrated that palbociclib induces G1 phase cell cycle arrest in HPV^−^ HNSCC cells, leading to reduced cell viability [5,6]. Our MTT assay results in CAL27 and HN5 cells confirmed this effect (Figure 1).

**Figure 2:**
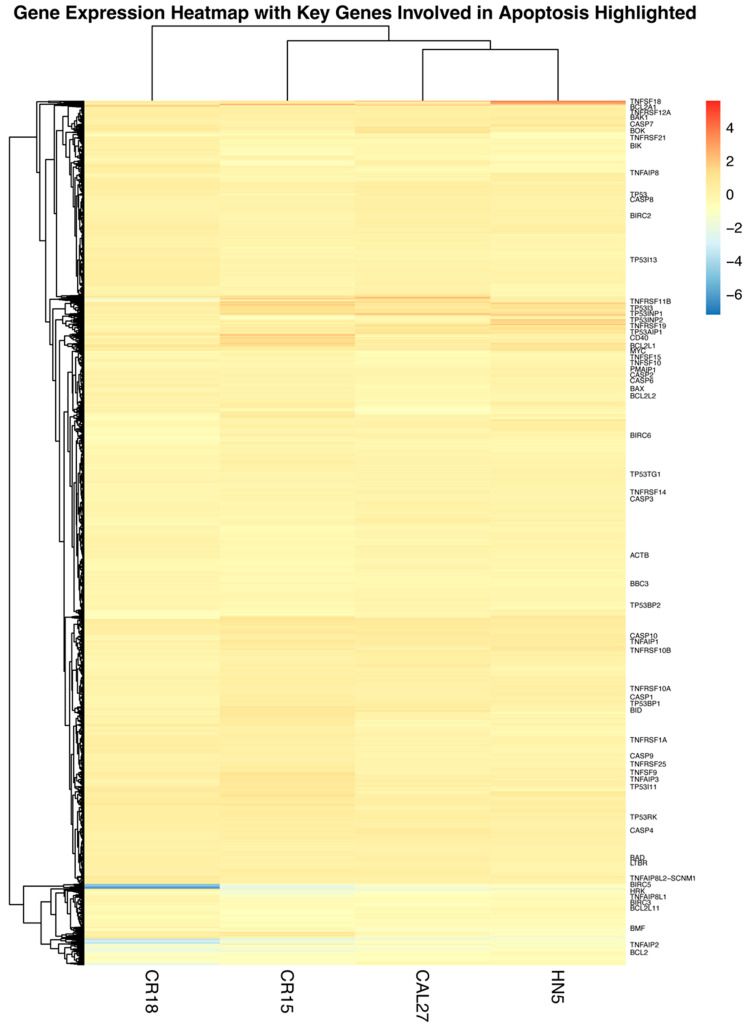
Differential expression heatmap. A heatmap visualizing differential gene expression (log2 fold change) was generated for the CAL27, CR15, CR18, and HN5 cell lines following palbociclib treatment. Key apoptosis-related genes are highlighted in the heatmap.

**Figure 3:**
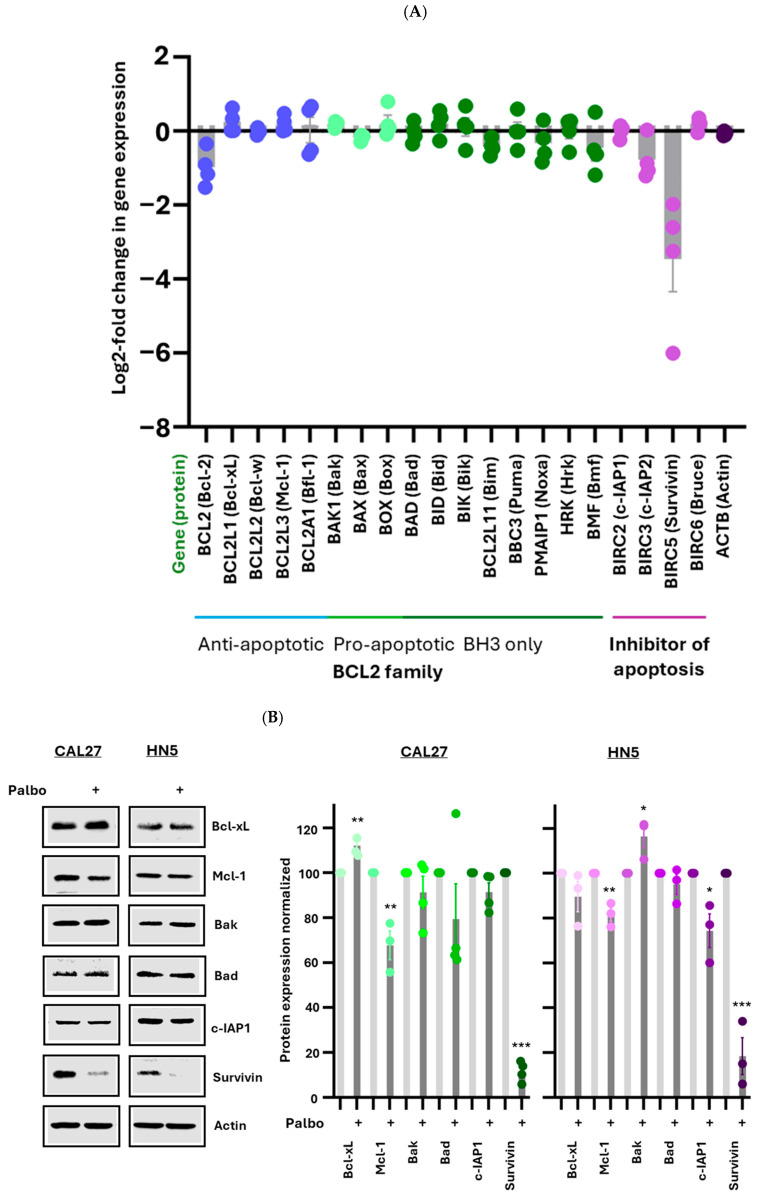
Effect of palbociclib on the expression of apoptosis-regulating proteins. (**A**) Gene expression analysis of apoptosis-regulating proteins. Each dot in the graph represents the expression of one gene in one cell type. A |Log2-fold change| > 1 indicates upregulation or downregulation relative to the control. (**B**) Protein expression analysis of apoptosis-regulating proteins: CAL27 and HN5 cells were treated with palbociclib (1 µM) or vehicle control for 72 h, followed by processing. Cell lysates were analyzed using Western blotting, with β-actin serving as the loading control. Full pictures of the Western blots are presented in Supplementary File. Bar graphs show the quantification of protein levels relative to actin control, and error bars represent the standard error of the mean. At least three independent experiments were performed. Analysis by two tailed t-test. * *p* < 0.05; ** *p* < 0.01; *** *p* < 0.001.

A decrease in cell viability may result from either reduced cell proliferation or increased cell death. To investigate whether palbociclib induces apoptosis, we performed RNA sequencing on CAL27, HN5, CR15, and CR18 cell lines, comparing treated cells with untreated controls. Differential transcriptomic profiling of cells following palbociclib treatment was visualized using a heatmap (Figure 2). Approximately 11,400 genes were detected across the four cell lines. Upon palbociclib treatment, all four lines exhibited a similar pattern in the log2 fold change in gene expression. We specifically examined the impact of palbociclib on the expression of two major families of apoptosis-regulating proteins: the BCL2 family and the inhibitor of apoptosis (IAP) family. The BCL2 family consists of 16 members, categorized into three groups: anti-apoptotic, pro-apoptotic, and pro-apoptotic BH3-only proteins. The IAP includes 8 members. Our RNA sequencing data revealed that, among the 16 BCL2 family members and 4 IAP family members detected, survivin showed the most significant decrease in mRNA levels following treatment with 1 µM palbociclib (Figure 3A).

Next, we selected one or two genes from each group to validate their protein expression in CAL27 and HN5. Western blot analysis demonstrated that palbociclib treatment led to the greatest reduction in survivin expression, with an approximately 88% decrease in CAL27 and about 84% in HN5 (Figure 3B). Although some of the other proteins also showed statistically significant decreases or increases, the magnitude of these changes was minimal compared to that of survivin (Figure 3B).

These findings indicate that, among all the apoptosis-regulating proteins analyzed, survivin is the only one affected dramatically by palbociclib. Given survivin’s dual role in both cell cycle progression and apoptosis inhibition, this result suggests that palbociclib may primarily suppress proliferation without inducing cell death. To explore this further, we assessed the expression level of cleaved caspase-3, a well-established marker of apoptosis, by Western blotting, following palbociclib treatment (1 µM, 72 h). Our results showed no increase in cleaved caspase-3 levels, indicating that under these conditions, CDK4/6 inhibition does not elicit a detectable apoptotic response.

Taken together, these results suggest that the reduced cell viability observed with palbociclib treatment is primarily due to impaired cell proliferation. Survivin may play a critical role in this process by regulating cell division.

### 3.2. Survivin Is Overexpressed in Human HNSCC Tissues

**Figure 4:**
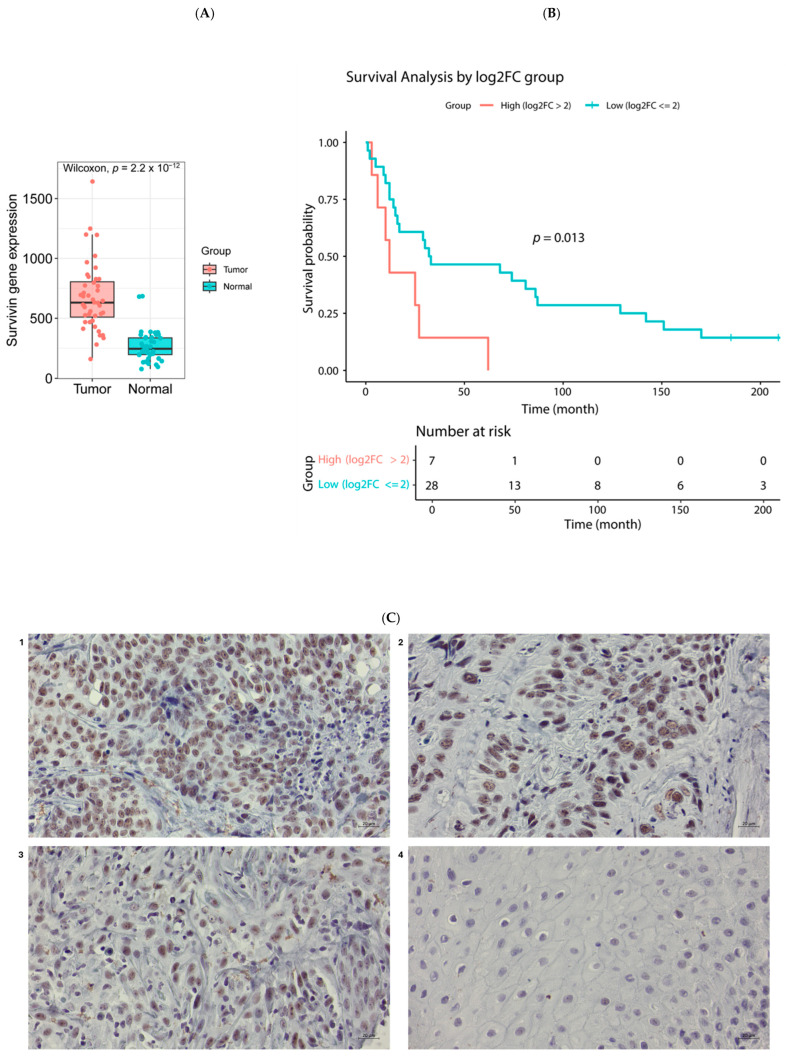
Survivin is over expressed in HNSCC. (**A**) Comparison of survivin gene expression: the gene expression of survivin in HNSCC was compared to normal tissues adjacent to the tumor. The bar graph shows the quantification of gene expression. (**B**) Kaplan–Meier curves generated using R (v4.4.1) show the association between survivin expression and overall survival in HNSCC. A |Log2-fold change| > 2 indicates a high survivin expression, whereas a |Log2-fold change| ≤ 2 indicates a low survivin expression. Higher survivin expression was associated with significantly worse overall survival in HNSCC. (**C**) Immunohistochemical analysis of paraffin-embedded human HNSCC cancer tissue slide using survivin antibody at dilution of 1:250 (under 40× lens). Representative images were shown. 1. HNSCC tissue section no. S12_24899 3E-3. 2. HNSCC tissue section no. SS23_24339 A1-3. 3. HNSCC tissue section no. SS13_35053 A1-3. 4. Normal tissues adjacent to the tumor: section no. SS17_18321 A1K-3. Scale bar represents 20 µm.

To investigate whether survivin expression differs between HNSCC and normal tissues, we analyzed RNA microarray data retrieved from the existing data repository in the Department of Pathology at the AECOM, which included 44 pairs of primary tumor and solid normal tissue samples. The expression of survivin was significantly higher in tumor tissues compared to normal tissues, with a log2-fold change of 1.346 and Wilcoxon paired test *p*-value: 2.16 × 10^−12^ (Figure 4A). Kaplan–Meier analysis further revealed that lower survivin expression was associated with better overall survival in HNSCC patients (Figure 4B).

Additionally, we conducted immunohistochemical analysis on tissue samples from 12 patients to evaluate survivin protein expression in HNSCC. Survivin expression was detected in 10 of the samples. Among these, survivin showed strong expression in 7 samples and weaker expression in the remaining 3. Notably, survivin was predominantly localized in the nucleus (Figure 4C).

### 3.3. Survivin Dimerization Inhibitor LQZ-7i Decreases Cell Proliferation

**Figure 5:**
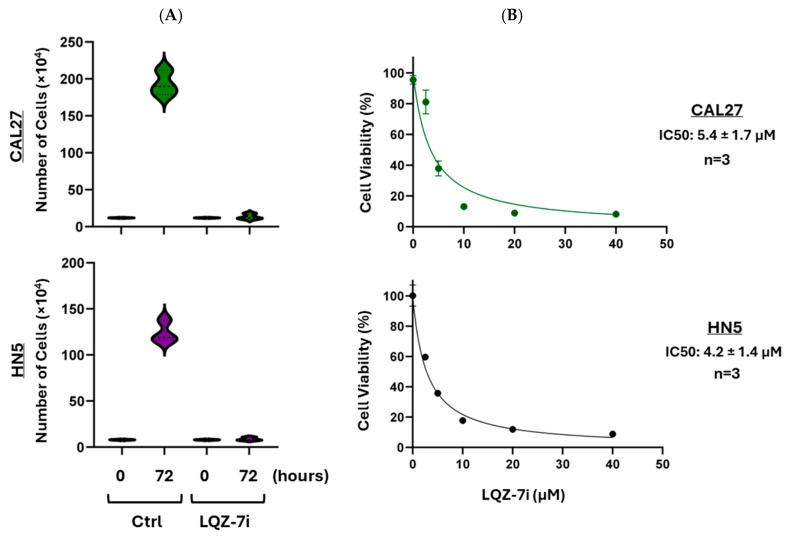
Effect of LQZ-7i on survivin protein levels and cancer cell viability. (**A**) Cell counting assay: CAL27 and HN5 cells were seeded in 6-well plates and treated with 7.5 µM LQZ-7i for 72 h. Cells were mixed with trypan blue and live cells were counted using a hemocytometer. The *X*-axis represents treatment times, and the *Y*-axis represents the number of cells per well. Three independent experiments were performed. (**B**) Cell viability assay: CAL27 and HN5 cells were seeded in 96-well plates and treated with LQZ-7i at specified concentrations for 72 h. An MTT assay was performed, and representative graphs are shown. The *x*-axis represents the drug concentration, and the *y*-axis represents the % cell viability. IC50 values represent the means ± SEM from three independent experiments conducted in triplicate.

To investigate the role of survivin in cancer cell proliferation, we treated CAL27 and HN5 cells with LQZ-7i, a novel and selective inhibitor of survivin dimerization [34]. Cell counting assays showed that, after three days of treatment, CAL27 cells treated with 7.5 µM LQZ-7i increased from 1.2 × 10^5^ to 1.3 × 10^5^ cells per well—a modest 1.1-fold increase. In contrast, untreated control cells grew from 1.2 × 10^5^ to 19.3 × 10^5^ cells, a 16-fold increase (Figure 5A). Similarly, HN5 cells treated with 7.5 µM LQZ-7i increased from 0.8 × 10^5^ to 0.9 × 10^5^ cells (1.1-fold), while control cells grew from 0.8 × 10^5^ to 12.1 × 10^5^ cells (15-fold) over the same period (Figure 5A). These results indicate that survivin inhibition markedly suppresses cell proliferation. Further analysis using the MTT assay demonstrated that LQZ-7i reduced cell viability in a dose-dependent manner, with IC50 values of 5.4 ± 1.7 µM for CAL27 and 4.2 ± 1.4 µM for HN5 cells (Figure 5B). Taken together, these findings suggest that reduced survivin dimerization contributes to decreased cell viability, reinforcing the role of survivin dimers in promoting tumor cell proliferation.

### 3.4. LQZ-7i Induces Cytoplasmic Localization of Survivin

**Figure 6:**
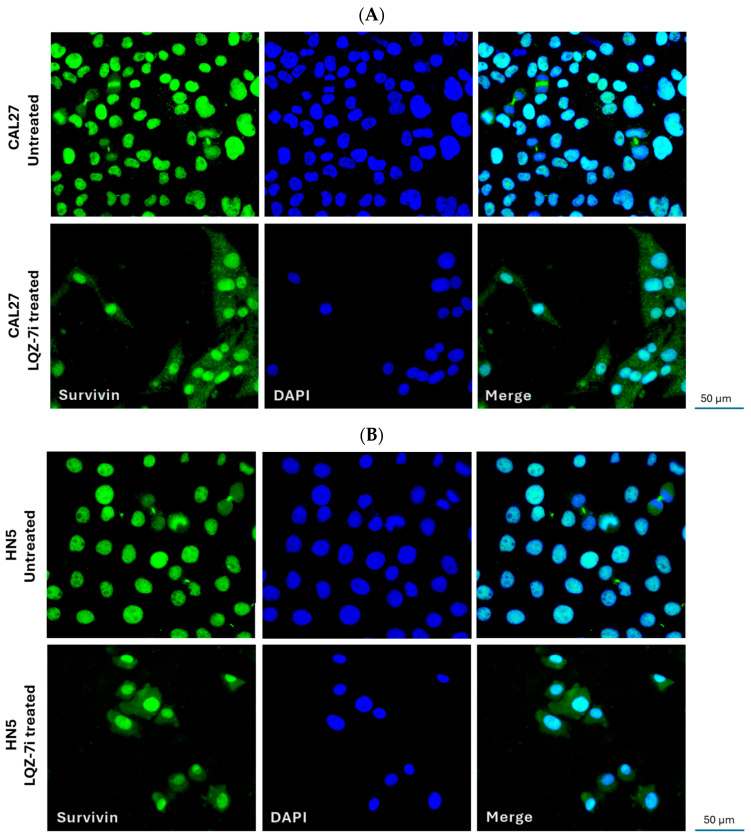
Effect of LQZ-7i on the subcellular localization of survivin. (**A**) Immunofluorescence staining of survivin in CAL27 cells. (**B**) Immunofluorescence staining of survivin in HN5 cells. CAL27 and HN5 cells were treated with 10 µM LQZ-7i or an equivalent volume of DMSO for 72 h. The cells were then fixed, permeabilized, and stained with an anti-survivin antibody (green). Nuclei were counterstained with DAPI (blue). Merged images are shown in the right panel. Scale bar = 50 µm. The morphology of fixed cells is similar to that of live cells. Three independent experiments were performed.

Survivin has been reported to localize in both the nucleus and cytoplasm of various cancer cells [35]. To explore this in HNSCC cells, we performed immunofluorescent staining to examine the subcellular localization of survivin. Fluorescent images showed that, in CAL27 and HN5 cells, survivin was predominantly localized in the nucleus (Figure 6A,B), consistent with the findings from tissue samples. However, following LQZ-7i treatment, we observed a shift in survivin’s subcellular localization, with a subset of survivin being exported from the nucleus to the cytoplasm (Figure 6A,B). This change in localization suggests that nuclear survivin may be associated with cell division whereas cytoplasmic survivin may contribute to cell survival by inhibiting apoptosis.

### 3.5. Palbociclib Suppresses USP1 Gene Expression

**Figure 7:**
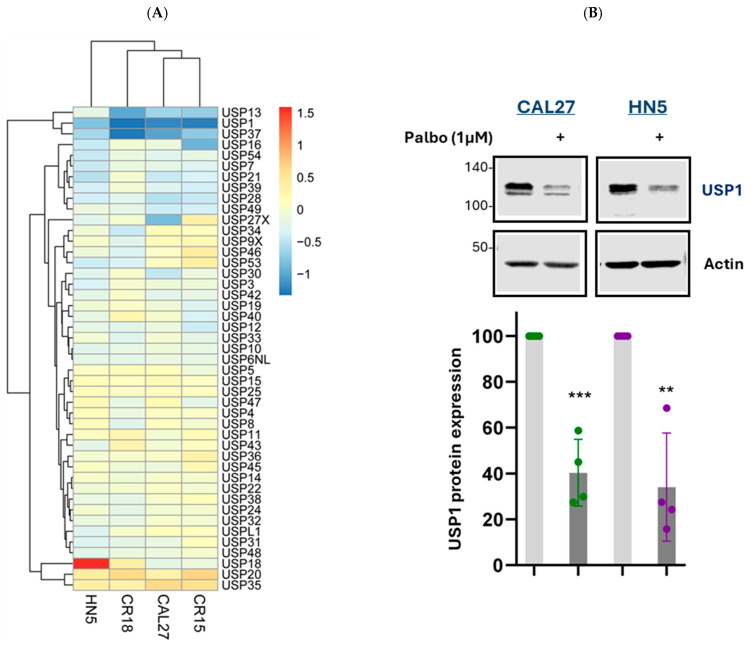
Effect of palbociclib on the expression of ubiquitin-specific peptidases. (**A**) Detection of gene expression of USP family members: CAL27, HN5, CR15, and CR18 cells were treated with palbociclib (1 µM) or vehicle control for 24 h. Total RNA was extracted, and differential gene expression was assessed through RNA-seq analysis, followed by a heatmap visualization. A |Log2-fold change| > 1 indicates up- or down-regulation relative to the control. (**B**) Detection of protein expression of USP1: CAL27 and HN5 cells were treated with palbociclib (1 µM) or vehicle control for 72 h. Cell lysates were analyzed by Western blotting, with β-actin used as the loading control. Full pictures of the Western blots are presented in Supplementary File. Bar graphs show the quantification of protein levels relative to actin control, and error bars represent the standard error of the mean. At least three independent experiments were performed. Analysis by two tailed *t*-test. ** *p* < 0.01; *** *p* < 0.001.

Survivin is ubiquitinated and degraded by the 26S proteasome [36]. Ubiquitin-specific peptidases (USPs), a family of 57 members, catalyze the removal of ubiquitin from ubiquitinated proteins, thereby stabilizing them by preventing degradation [37]. To identify which USP family member is the downstream target of CDK4/6, we investigated the effect of palbociclib on the gene expression of the USP family. Our RNA sequencing data revealed that, among the 45 USP family members detected, USP1 exhibited the most significant decrease in mRNA levels following treatment with 1 µM palbociclib (Figure 7A).

The protein expression of USP1 was further validated. Western blot analysis showed that palbociclib treatment led to a substantial reduction in USP1 protein levels, with an approximately 60% decrease in CAL27 cells and about 65% in HN5 cells (Figure 7B). These findings suggest that USP1 is a downstream target of CDK4/6.

### 3.6. Survivin as a Substrate of USP1

**Figure 8:**
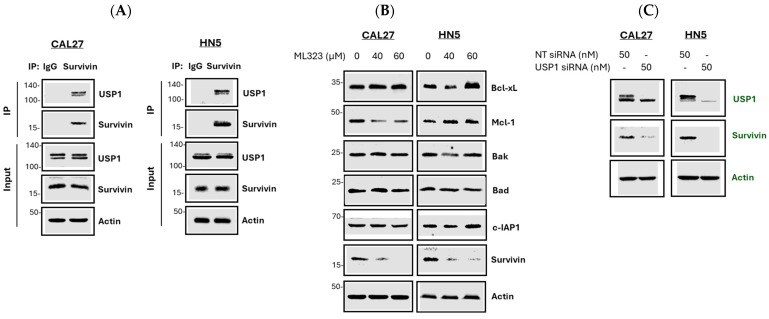
Examination of the interaction between USP1 and survivin. (**A**) Analysis of interactions between endogenous USP1 and survivin in CAL27 and HN5 cells: cell lysates were immunoprecipitated with survivin-specific antibodies and immunoblotted with the indicated antibodies. Experiments were conducted in duplicate. (**B**) Effect of USP1 inhibitor on survivin and other apoptosis-regulating proteins: CAL27 and HN5 cells were treated with ML323 at the indicated concentrations for 24 h, and cell lysates were analyzed by Western blotting with the indicated antibodies. Experiments were conducted in duplicate. (**C**) Effect of USP1 knockdown on survivin expression: CAL27 and HN5 cells were transfected with either non-targeting (NT) siRNA or USP1 siRNA for 24 h. Protein expression levels were measured by Western blotting. Experiments were conducted in triplicate. Full pictures of the Western blots are presented in Supplementary File.

To investigate whether survivin is a substrate of USP1, we performed co-immunoprecipitation to assess the interaction between the two proteins. Our results demonstrated that USP1 was co-precipitated with survivin (Figure 8A), indicating an interaction between them. Moreover, the inhibition of USP1 activity using its specific inhibitor ML323 resulted in a reduction in survivin protein levels in both CAL27 and HN5 cells (Figure 8B). In contrast, the protein levels of other apoptosis-regulating proteins such as Bcl-xL, Mcl-1, Bak, Bad, and c-IAP1 showed minimal, or no changes compared to survivin (Figure 8B). Additionally, the siRNA-mediated knockdown of USP1 led to decreased survivin protein levels in both CAL27 and HN5 cells (Figure 8C). These findings suggest that survivin is a substrate of USP1.

### 3.7. Inhibition of USP1 Reduces Survivin Protein Levels and Decreases Cell Viability

**Figure 9:**
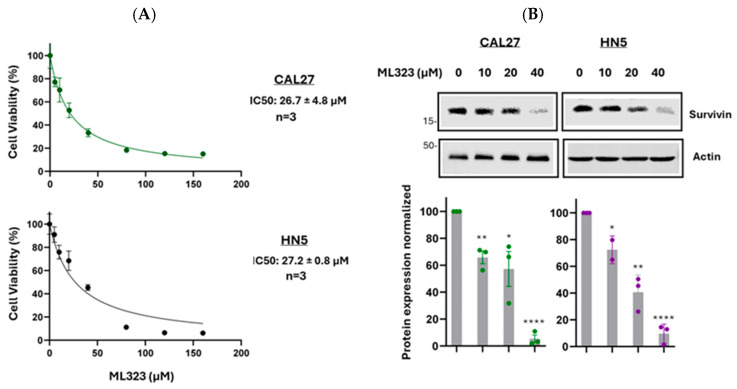
Effect of ML323 on survivin protein levels and cancer cell proliferation. (**A**) Detection of cell proliferation: CAL27 and HN5 cells were seeded in 96-well plates and treated with ML323 at the indicated concentrations for 72 h. An MTT assay was performed, and representative graphs are shown. The *x*-axis represents the drug concentration, and the *y*-axis represents the % cell viability. IC50 values represent the means ± SEM from three independent experiments performed in triplicate. (**B**) Detection of survivin protein expression: CAL27 and HN5 cells were cultured in the presence of ML323 at the indicated concentrations for 72 h. Cell lysates were analyzed by Western blotting, with actin used as the loading control. Full pictures of the Western blots are presented in Supplementary File. Data are presented as mean ± SEM from three independent experiments. Analysis by ANOVA. * *p* < 0.05; ** *p* < 0.01; **** *p* < 0.0001.

We next examined whether inhibiting USP1 impacts cell proliferation. The MTT as-say showed that ML323 inhibited cell viability in a dose-dependent manner, with IC50 values of 26.7 ± 4.8 µM for CAL27 and 27.2 ± 0.8 µM for HN5 cells (Figure 9A). We then assessed the effect of ML323 on survivin protein levels. Western blot analysis demonstrated that ML323 treatment reduced survivin protein levels in a dose-dependent manner, with an approximately 95% reduction in CAL27 cells and about a 90% reduction in HN5 cells at a concentration of 40 µM (Figure. 9B). These results suggest that the reduction in survivin expression correlates with decreased cell viability, highlighting the role of survivin in tumor cell proliferation.

## 4. Discussion

### 4.1. Elevated Survivin Levels in HNSCC

Our study found that survivin is overexpressed in HNSCC at both the mRNA and protein levels. Muzio et al. [38] examined survivin protein expression in 110 HNSCC cases, including six lymph node and one distant metastatic lesion, using immunohistochemistry and Western blotting. They found that 91 of these cases (82.7%) and all seven metastatic cases (100%) were positive for survivin expression. Additionally, survivin gene expression was significantly increased in tumor tissues across 33 cancer types based on RNA sequencing data from the TCGA database [39,40]. Immunohistochemistry analysis of survivin expression in both tumor and normal tissues from the Human Protein Atlas revealed a similar pattern [40]. Prognostic analysis of survivin expression across 33 cancer types in the TCGA database indicated that for 14 of these types, higher survivin expression was associated with poorer overall survival, suggesting that survivin could serve as a promising prognostic biomarker for these cancers [39]. Several meta-analyses of HNSCC have also indicated that higher survivin expression correlates with poorer overall survival [38,41,42]. Our data similarly showed that increased survivin expression in HNSCC was associated with worse overall survival, indicating that survivin expression may help identify HNSCC cases with more aggressive and invasive phenotypes.

### 4.2. Subcellular Localization of Survivin and Its Biological Function

We observed that survivin was predominantly localized in the nucleus of HPV^−^ HNSCC cells. Treatment with LQZ-7i, a compound that disrupts survivin dimerization, significantly reduced cell viability, underscoring the role of nuclear survivin in supporting cell proliferation. Interestingly, LQZ-7i also caused a portion of survivin to relocalize from the nucleus to the cytoplasm. This may be due to the ability of monomeric survivin—but not its dimeric form—to pass through the nuclear pore complex. Alternatively, monomeric survivin might be transported to the cytoplasm under stress conditions to exert cytoprotective effects and counteract apoptosis. These observations support the idea that nuclear survivin primarily regulates cell division, while cytoplasmic survivin may function to inhibit cell death [43,44,45]. During mitosis, survivin localizes to the mitotic spindle, where it interacts with microtubules to organize the spindle and assist in chromosome segregation [19]. Survivin is exported from the nucleus in an exportin-1 (also known as Crm1)-dependent manner, with a nuclear export sequence (NES) located between the BIR domain and C-terminal helix [46,47]. This NES is exposed in the survivin monomer but masked in the dimer, which prevents its nuclear export. Mutants of survivin that do not dimerize exhibit increased cytoplasmic localization, suggesting that dimerization inhibits nuclear export [10,48]. We demonstrated that disrupting survivin dimerization with LQZ-7i promoted its export from the nucleus to the cytoplasm. Although the exact regulatory mechanisms behind survivin localization remain unclear, our findings support the idea that survivin’s subcellular location—whether in the cytoplasm or nucleus—affects its function. Our data also suggest that the dimeric or monomeric state of survivin is crucial for its intracellular trafficking, and targeting factors that govern the dimer-monomer transition could direct survivin to specific subcellular locations.

### 4.3. Regulation of Survivin by Ubiquitin-Specific Peptidase 1 in HNSCC Cells

USP1 is a member of the cysteine protease family and functions by hydrolyzing the isopeptide bond that connects ubiquitin to its target protein or to other ubiquitin molecules within a polyubiquitin chain. Its catalytic activity is driven by a triad of conserved amino acids—Cys90, His593, and Asp751—located within the Cys and His boxes [49]. USP1 requires interacting with its cofactor UAF1 to achieve full enzymatic activity [50].

Well-characterized substrates of USP1 include FANCD2 and FANCI, key components of the Fanconi anemia DNA repair pathway, as well as PCNA, which plays a central role in DNA replication and repair. Arkinson et al. [51] demonstrated that the N-terminal region of USP1 contains a FANCD2-specific binding sequence essential for deubiquitinating FANCD2 at Lys561. Dharadhar et al. [52] identified that Insert L1 and the region spanning amino acids 420–520 of USP1 are crucial for interacting with DNA-bound ubiquitinated PCNA and for binding to UAF1, respectively.

Our findings indicate that survivin is a substrate of USP1 in HNSCC cells. Supporting this, Woo et al. [53] reported that survivin directly binds to USP1 in Caki-1 cells. The overexpression of wild-type USP1 significantly reduced survivin ubiquitination, while the expression of a catalytically inactive mutant (C90S) led to increased ubiquitination. These results suggest that USP1 actively cleaves ubiquitin from survivin.

Survivin is known to be polyubiquitinated during mitosis through both Lys48- and Lys63-linked chains [54]. Lys48 linkages typically signal for proteasomal degradation, whereas Lys63 linkages are involved in various signaling pathways. USP1/UAF1 has been shown to cleave both Lys48- and Lys63-linked ubiquitin chains on PCNA, primarily through an exo-cleavage mechanism that trims the polyubiquitin chain before complete removal [55]. Whether USP1/UAF1 exhibits similar cleavage activity on survivin’s ubiquitin chains, and the precise mechanism by which USP1 recognizes and binds survivin, remain unknown. Further studies are needed to elucidate these molecular details.

### 4.4. Regulation of Survivin by the CDK4/6-Rb Pathway

Our data revealed that the mRNA expression of survivin in HPV^−^ HNSCC cells was downregulated by palbociclib, an inhibitor of CDK4/6, indicating that the CDK4/6-Rb pathway regulates survivin gene expression. Similar results have been observed in other cancer types [56]. CDK4/6 is a key regulator of the cell cycle that forms a complex with cyclin D1, which phosphorylates and inactivates the Rb protein. This release of E2F transcription factors drives cell proliferation [27,57]. E2F activators (E2F1, E2F2, and E2F3) can bind to the survivin promoter and enhance its transcription, while genetic loss of E2F activators inhibits survivin expression [58,59]. Palbociclib disrupts CDK4/6-cyclin D1 complexes, preventing Rb phosphorylation and blocking E2F activation, leading to the repression of E2F target genes, including survivin, causing G1 phase arrest and the suppression of cell proliferation.

### 4.5. Palbociclib Effects on HPV-Negative and HPV-Positive HNSCC

We demonstrate that palbociclib suppresses cell proliferation in HPV^−^ HNSCC cell lines, specifically CAL27 and HN5. Treated cells also exhibit notable morphological changes, including an increased cell size and a flattened appearance—hallmarks of cellular senescence. These findings are consistent with previous studies.

For example, Gadsden et al. [5] reported that HPV^−^ cell lines (CAL27, HN31, PCI15B) treated with palbociclib show inhibited cell division and enlarged morphology compared to untreated controls. In contrast, HPV+ cell lines (UM-SCC-47, UPCI-SCC-090, UPCI-SCC-154) displayed minimal changes in both proliferation and morphology following treatment. Similarly, Gottgens et al. [60] found that palbociclib induces senescence and may enhance radiosensitivity in HPV^−^ HNSCC by impairing DNA damage repair pathways. This effect was largely absent in HPV+ cells, indicating a differential response based on HPV status. Collectively, these observations suggest that palbociclib may serve as a promising therapeutic strategy in HPV^−^ HNSCC.

Despite this potential, palbociclib has shown limited efficacy as a monotherapy in HPV^−^ HNSCC, likely due to the complexity of its mechanisms. However, when used in combination with other therapies, palbociclib shows enhanced effectiveness.

In HPV^−^ HNSCC, palbociclib has been shown to enhance the efficacy of radiation therapy by inhibiting the repair of radiation-induced DNA damage, resulting in increased tumor cell death [6,61]. Additionally, it exhibits synergistic effects when combined with inhibitors targeting PI3K (e.g., alpelisib), EGFR (e.g., cetuximab), and MEK (e.g., selumetinib) pathways [56,62]. Furthermore, palbociclib-induced senescence makes tumor cells more vulnerable to senolytic agents such as navitoclax, which selectively eliminate senescent cells [6].

Our work further highlights the role of survivin, which is overexpressed in HPV^−^ HNSCC and regulated by the deubiquitinase USP1. Although initially classified as an inhibitor of apoptosis, recent research indicates that survivin primarily functions in mitotic regulation. The disruption of survivin can lead to mitotic catastrophe, while its overexpression enables tumor cells to bypass cell cycle checkpoints and resist cell death [63]. This suggests that elevated survivin levels may contribute to resistance against palbociclib by supporting continued cell division and survival.

USP1 also plays a critical role in DNA repair by deubiquitinating key proteins such as FANCD2 and PCNA, thereby regulating the Fanconi anemia pathway and homologous recombination [51,52]. Additionally, USP1 stabilizes survivin, further promoting cell survival and resistance to apoptosis [53]. Alterations in USP1 activity could therefore compromise the efficacy of palbociclib by enabling tumor cells to evade CDK4/6-mediated cell cycle arrest or by improving DNA repair capacity.

Given these insights, targeting survivin or USP1 may enhance the therapeutic impact of palbociclib in HPV^−^ HNSCC. Our findings suggest that combination strategies involving palbociclib and inhibitors of survivin or USP1 hold potential for overcoming resistance mechanisms. Further investigation and clinical validation are warranted to optimize palbociclib-based treatment regimens, particularly for HPV^−^ HNSCC.

## 5. Conclusions

**Figure 10:**
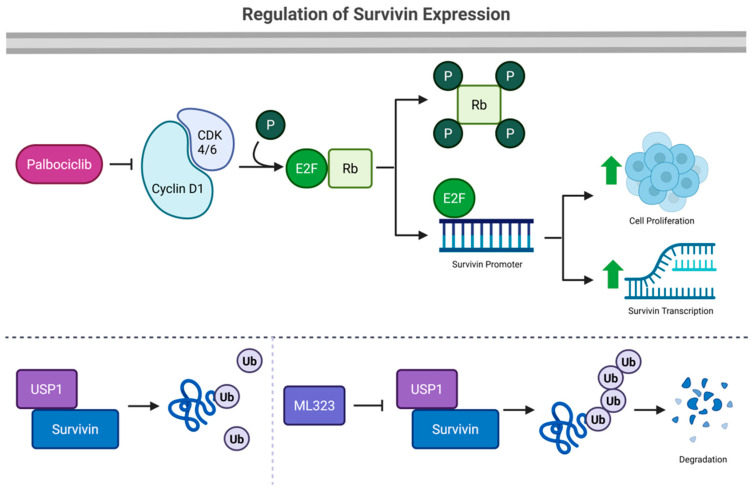
Schematic illustrating the regulation of survivin expression. Upper panel: CDK4/6 binds to cyclin D1, forming active cyclin D1–CDK4/6 complexes. These active complexes phosphorylate the Rb protein, allowing the release of the E2F transcription factor. The free E2F further binds to the survivin gene promotor and promotes its transcription, contributing to enhanced cell proliferation. Palbociclib inhibits CDK4/6 activity, resulting in decreased survivin expression and reduced cell proliferation. Lower panel: USP1 interacts with survivin and induces the deubiquitination of survivin. When USP1 is inhibited by ML323, survivin is ubiquitinated and degraded.

Our findings identify survivin as a key driver of cell proliferation in HPV^−^ HNSCC. Inhibiting survivin effectively suppresses cell proliferation, while the activation of CDK4/6 and USP1 promotes its upregulation (Figure 10). These results suggest that targeting survivin directly—or interfering with its upstream regulators—may enhance the therapeutic impact of CDK4/6 inhibition in this cancer subtype.

## Data Availability

The data presented in this study are available on request from the corresponding author. Research data are stored in the lab repository and will be shared upon request to the corresponding author.

## Supplementary Materials

The following supporting information can be downloaded at: https://www.mdpi.com/article/10.3390/cancers17172864/s1. File S1: full-length Western blot images.

## Abbreviations

The following abbreviations are used in this manuscript:

HNSCC	Head and neck squamous cell carcinoma
HPV	Human papillomavirus
CDK4/6	Cyclin-dependent kinase 4 and 6
IAP	Inhibitor of apoptosis proteins
USP1	Ubiquitin specific peptidase 1
