# Supplementary material for: Survivin Is a Central Mediator of Cell Proliferation in HPV-Negative Head and Neck Squamous Cell Carcinoma
Source: Cancers (Basel). 2025 Aug 31;17(17):2864. doi: 10.3390/cancers17172864 (PMC12427275; doi:10.3390/cancers17172864)
Supplement: Supplementary file 1 [file cancers-17-02864-s001.zip › File S1. Full-lengthWestern blot images/Original whole blot Fig7B.pptx]

## Slide 1
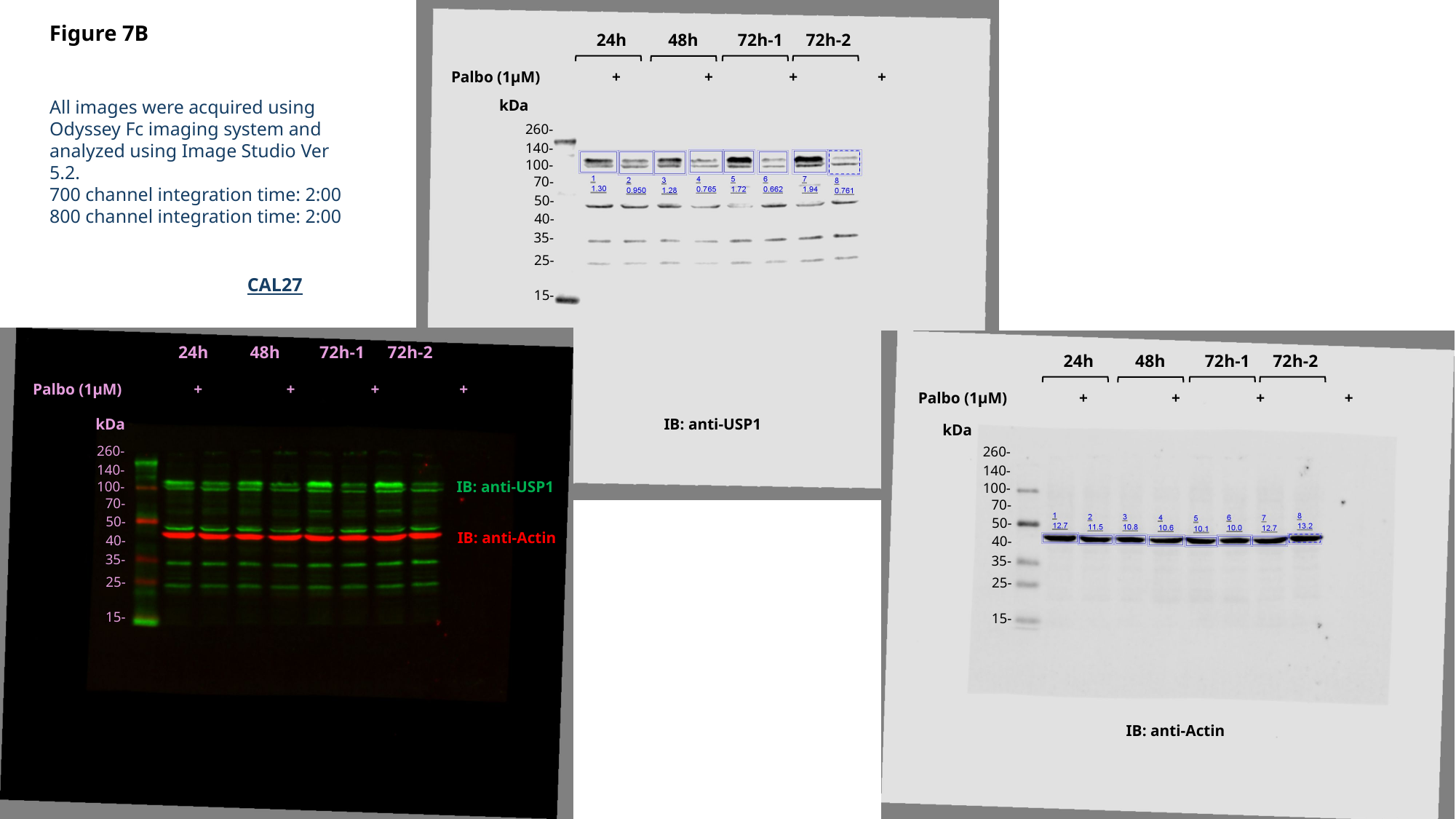

24h
72h-1
72h-2
48h
Palbo (1µM) + + + +
kDa
260-
140-
100-
70-
50-
40-
35-
25-
15-
IB: anti-USP1
Figure 7B
All images were acquired using Odyssey Fc imaging system and analyzed using Image Studio Ver 5.2.
700 channel integration time: 2:00
800 channel integration time: 2:00
CAL27
24h
72h-1
72h-2
48h
Palbo (1µM) + + + +
kDa
260-
140-
100-
70-
50-
40-
35-
25-
15-
IB: anti-Actin
24h
72h-1
72h-2
48h
Palbo (1µM) + + + +
kDa
260-
140-
100-
70-
50-
40-
35-
25-
15-
IB: anti-USP1
IB: anti-Actin

## Slide 2
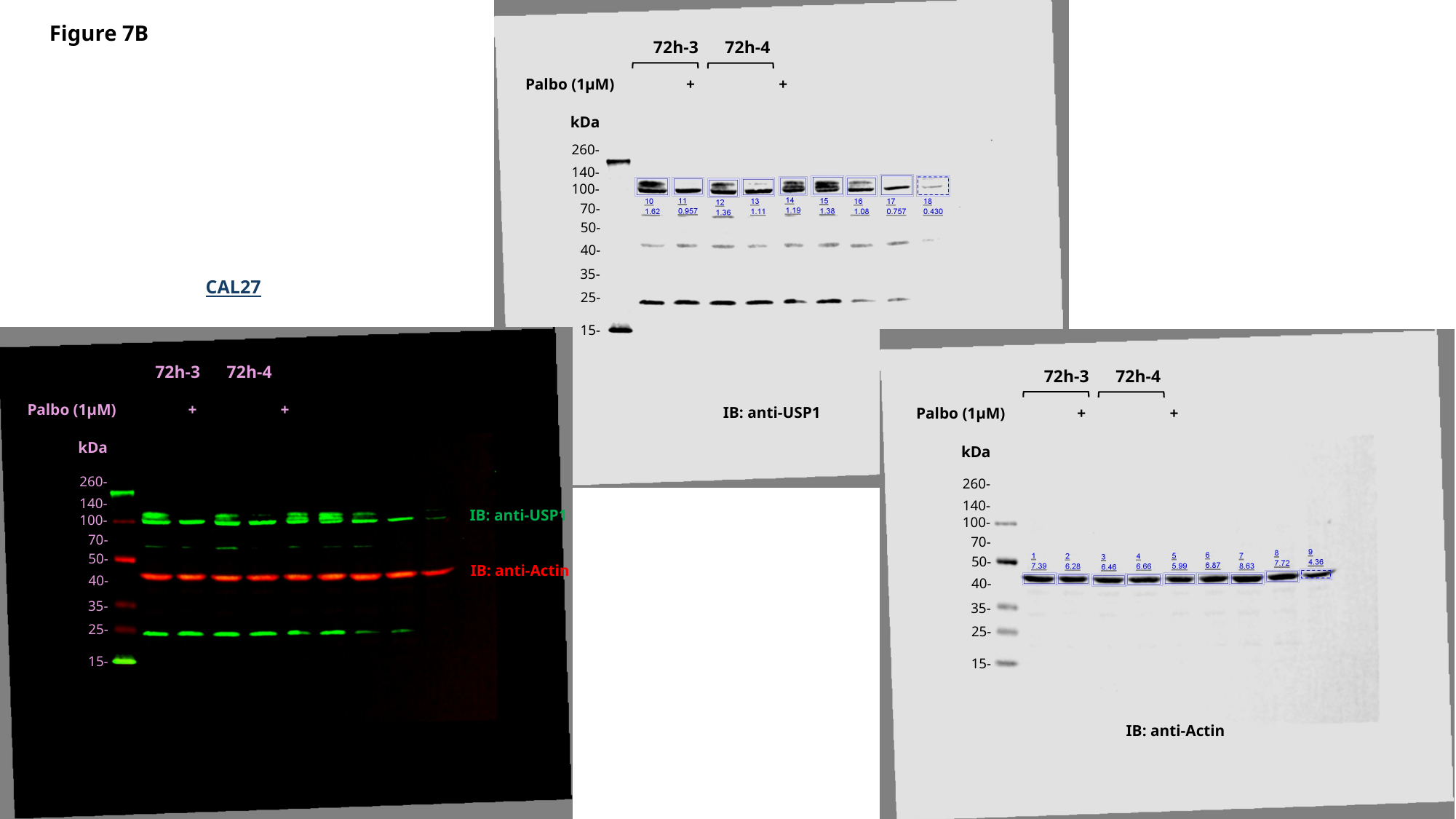

72h-3
72h-4
Palbo (1µM) + +
kDa
260-
140-
100-
70-
50-
40-
35-
25-
15-
IB: anti-USP1
Figure 7B
CAL27
72h-3
72h-4
Palbo (1µM) + +
kDa
260-
140-
100-
70-
50-
40-
35-
25-
15-
IB: anti-USP1
IB: anti-Actin
72h-3
72h-4
Palbo (1µM) + +
kDa
260-
140-
100-
70-
50-
40-
35-
25-
15-
IB: anti-Actin

## Slide 3
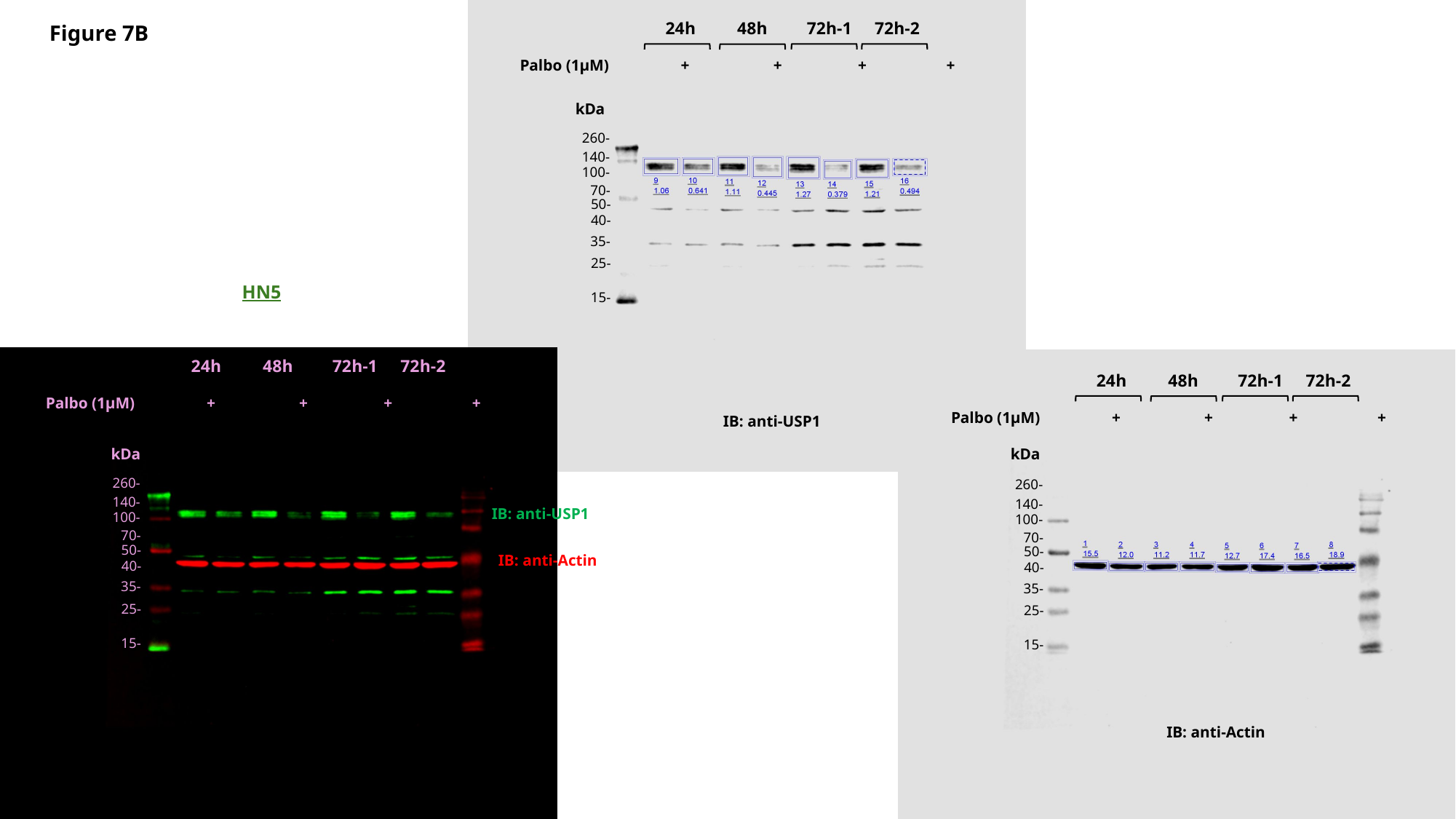

24h
72h-1
72h-2
48h
Palbo (1µM) + + + +
kDa
260-
140-
100-
70-
50-
40-
35-
25-
15-
IB: anti-USP1
Figure 7B
HN5
24h
72h-1
72h-2
48h
Palbo (1µM) + + + +
kDa
260-
140-
100-
70-
50-
40-
35-
25-
15-
IB: anti-USP1
IB: anti-Actin
24h
72h-1
72h-2
48h
Palbo (1µM) + + + +
kDa
260-
140-
100-
70-
50-
40-
35-
25-
15-
IB: anti-Actin

## Slide 4
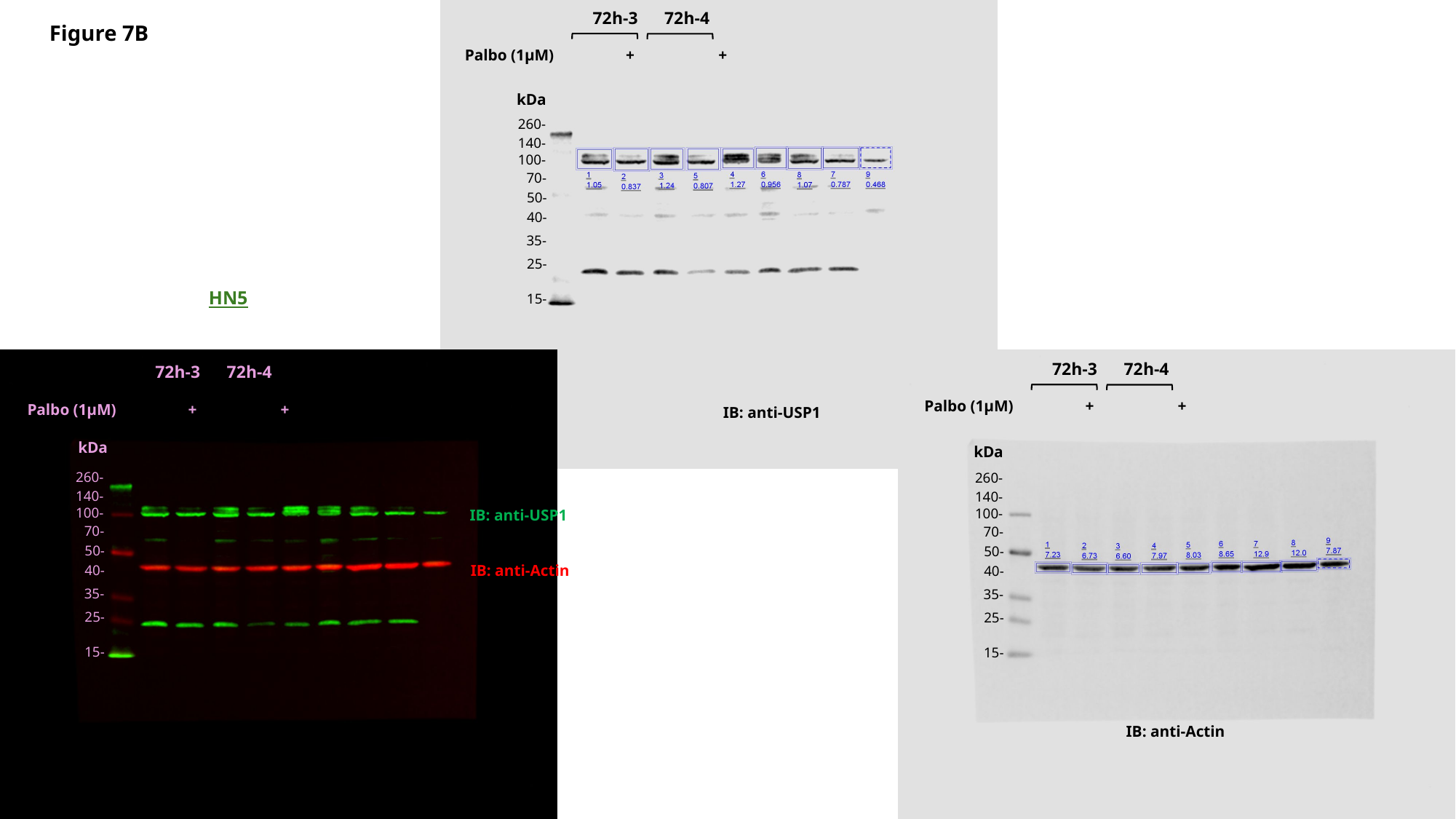

72h-3
72h-4
Palbo (1µM) + +
kDa
260-
140-
100-
70-
50-
40-
35-
25-
15-
IB: anti-USP1
Figure 7B
HN5
72h-3
72h-4
Palbo (1µM) + +
kDa
260-
140-
100-
70-
50-
40-
35-
25-
15-
IB: anti-USP1
IB: anti-Actin
72h-3
72h-4
Palbo (1µM) + +
kDa
260-
140-
100-
70-
50-
40-
35-
25-
15-
IB: anti-Actin
