# Supplementary material for: Survivin Is a Central Mediator of Cell Proliferation in HPV-Negative Head and Neck Squamous Cell Carcinoma
Source: Cancers (Basel). 2025 Aug 31;17(17):2864. doi: 10.3390/cancers17172864 (PMC12427275; doi:10.3390/cancers17172864)
Supplement: Supplementary file 1 [file cancers-17-02864-s001.zip › File S1. Full-lengthWestern blot images/Original whole blot Fig8C.pptx]

## Slide 1
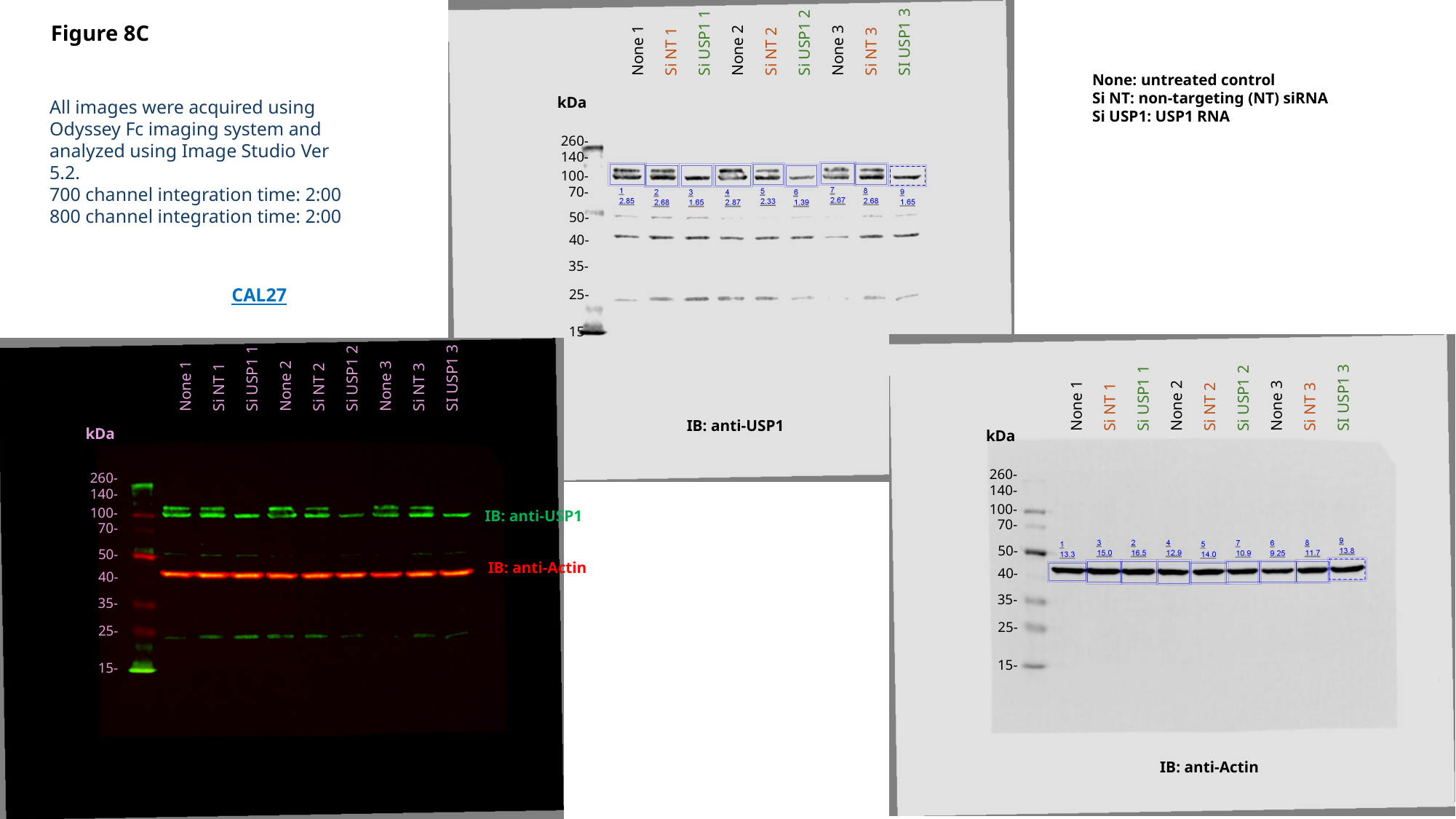

None 1
Si NT 1
Si USP1 1
None 2
Si NT 2
Si USP1 2
None 3
Si NT 3
SI USP1 3
kDa
260-
140-
100-
70-
50-
40-
35-
25-
15-
IB: anti-USP1
Figure 8C
None: untreated control
Si NT: non-targeting (NT) siRNA
Si USP1: USP1 RNA
All images were acquired using Odyssey Fc imaging system and analyzed using Image Studio Ver 5.2.
700 channel integration time: 2:00
800 channel integration time: 2:00
CAL27
None 1
Si NT 1
Si USP1 1
None 2
Si NT 2
Si USP1 2
None 3
Si NT 3
SI USP1 3
kDa
260-
140-
100-
70-
50-
40-
35-
25-
15-
IB: anti-Actin
None 1
Si NT 1
Si USP1 1
None 2
Si NT 2
Si USP1 2
None 3
Si NT 3
SI USP1 3
kDa
260-
140-
100-
70-
50-
40-
35-
25-
15-
IB: anti-USP1
IB: anti-Actin

## Slide 2
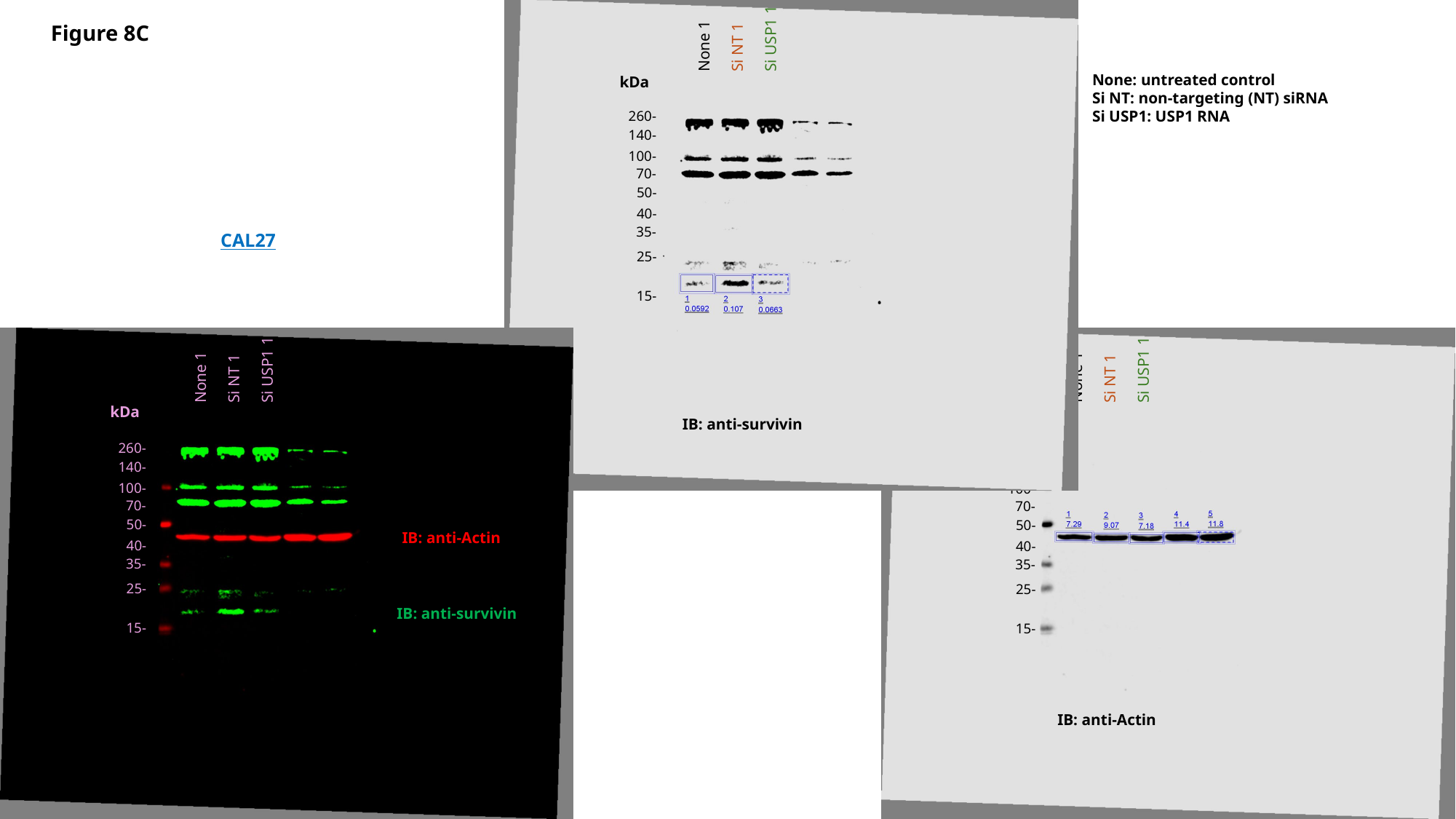

None 1
Si NT 1
Si USP1 1
kDa
260-
140-
100-
70-
50-
40-
35-
25-
15-
IB: anti-survivin
Figure 8C
None: untreated control
Si NT: non-targeting (NT) siRNA
Si USP1: USP1 RNA
CAL27
None 1
Si NT 1
Si USP1 1
kDa
260-
140-
100-
70-
50-
40-
35-
25-
15-
IB: anti-Actin
IB: anti-survivin
None 1
Si NT 1
Si USP1 1
kDa
260-
140-
100-
70-
50-
40-
35-
25-
15-
IB: anti-Actin

## Slide 3
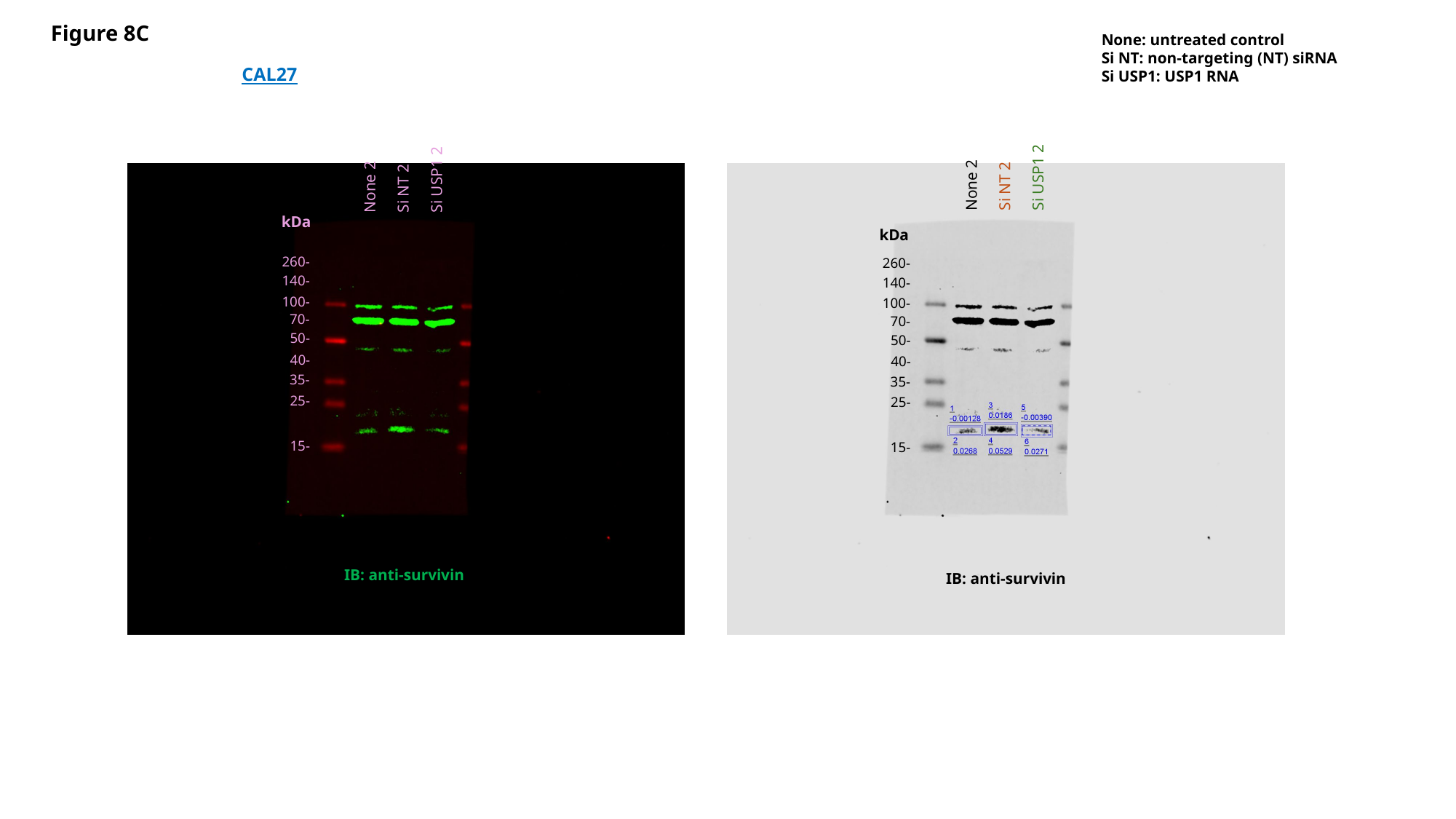

Figure 8C
None: untreated control
Si NT: non-targeting (NT) siRNA
Si USP1: USP1 RNA
CAL27
None 2
Si NT 2
Si USP1 2
kDa
260-
140-
100-
70-
50-
40-
35-
25-
15-
IB: anti-survivin
None 2
Si NT 2
Si USP1 2
kDa
260-
140-
100-
70-
50-
40-
35-
25-
15-
IB: anti-survivin

## Slide 4
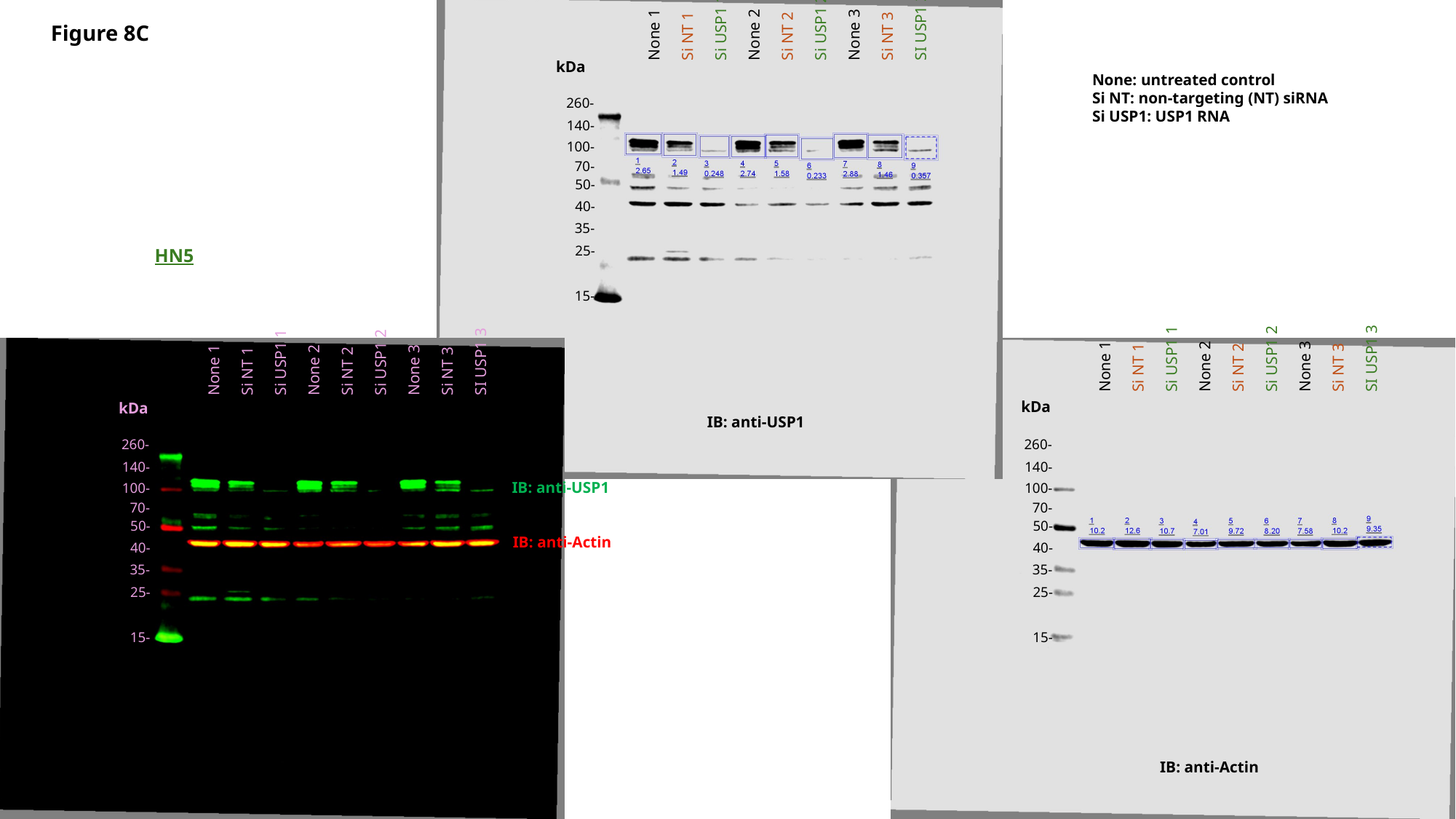

None 1
Si NT 1
Si USP1 1
None 2
Si NT 2
Si USP1 2
None 3
Si NT 3
SI USP1 3
kDa
260-
140-
100-
70-
50-
40-
35-
25-
15-
IB: anti-USP1
Figure 8C
None: untreated control
Si NT: non-targeting (NT) siRNA
Si USP1: USP1 RNA
HN5
None 1
Si NT 1
Si USP1 1
None 2
Si NT 2
Si USP1 2
None 3
Si NT 3
SI USP1 3
kDa
260-
140-
100-
70-
50-
40-
35-
25-
15-
IB: anti-Actin
None 1
Si NT 1
Si USP1 1
None 2
Si NT 2
Si USP1 2
None 3
Si NT 3
SI USP1 3
kDa
260-
140-
100-
70-
50-
40-
35-
25-
15-
IB: anti-USP1
IB: anti-Actin

## Slide 5
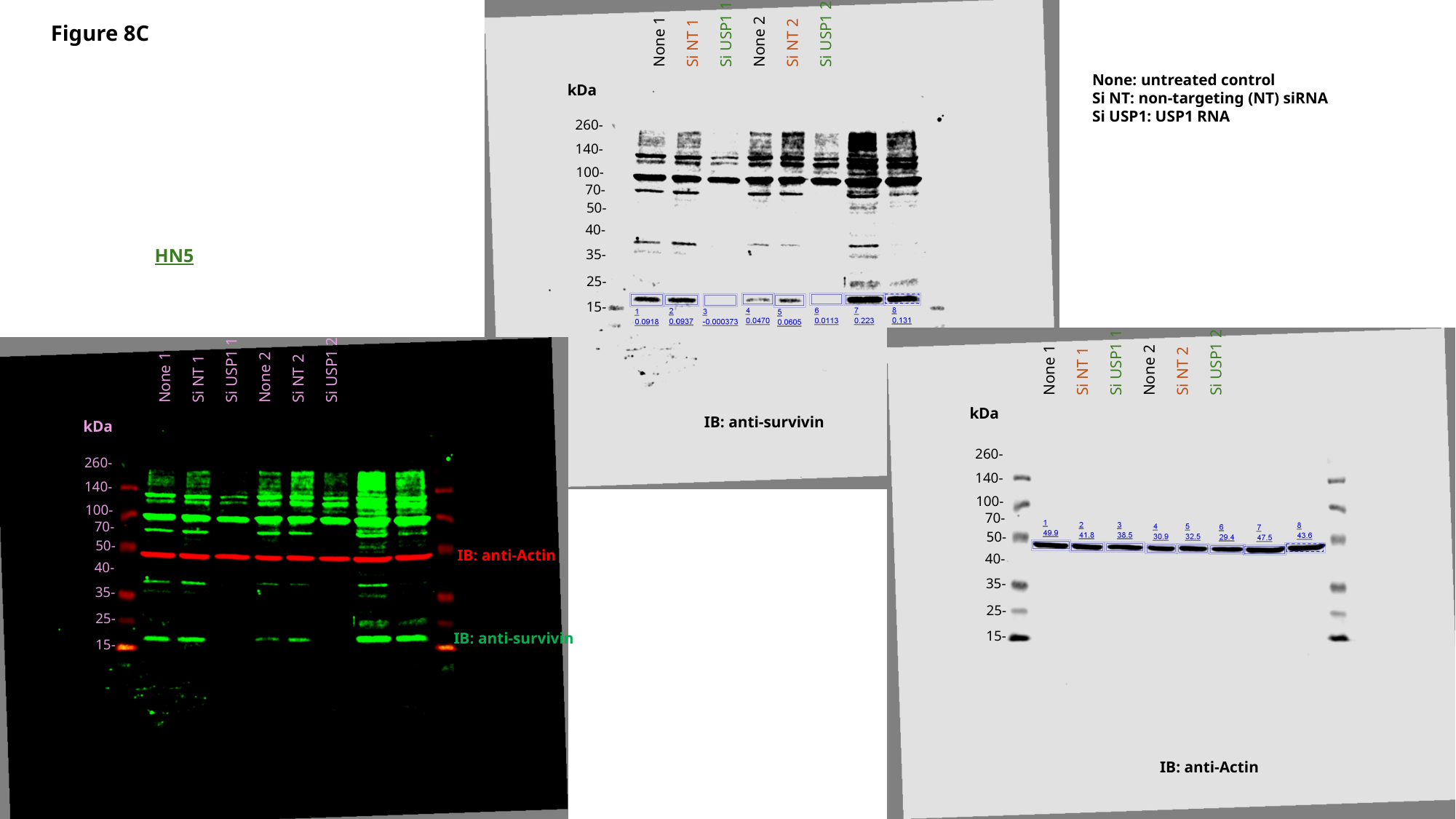

None 1
Si NT 1
Si USP1 1
None 2
Si NT 2
Si USP1 2
kDa
260-
140-
100-
70-
50-
40-
35-
25-
15-
IB: anti-survivin
Figure 8C
None: untreated control
Si NT: non-targeting (NT) siRNA
Si USP1: USP1 RNA
HN5
None 1
Si NT 1
Si USP1 1
None 2
Si NT 2
Si USP1 2
kDa
260-
140-
100-
70-
50-
40-
35-
25-
15-
IB: anti-Actin
None 1
Si NT 1
Si USP1 1
None 2
Si NT 2
Si USP1 2
kDa
260-
140-
100-
70-
50-
40-
35-
25-
15-
IB: anti-Actin
IB: anti-survivin
